# Medical Students’ Attitudes, Knowledge, and Beliefs about Medical Cannabis: A Qualitative Descriptive Study

**DOI:** 10.7759/cureus.28336

**Published:** 2022-08-24

**Authors:** Robin J Jacobs, Jessica Colon, Michael N Kane

**Affiliations:** 1 Medical and Behavioral Research, Health Informatics, and Medical Education, Nova Southeastern University, Fort Lauderdale, USA; 2 Medical School, Nova Southeastern University Dr. Kiran C. Patel College of Osteopathic Medicine, Fort Lauderdale, USA; 3 Social Work, Florida Atlantic University, Boca Raton, USA

**Keywords:** medical education curriculum, focus groups, qualitative, beliefs, attitudes, education, medical marijuana, medical cannabis, medical students

## Abstract

Background

There has been increased attention given to understanding the uses of medical cannabis (MC) for symptom management of various medical conditions. Physicians receive minimal training in medical school and rely mostly on anecdotal evidence; by proxy, medical students generally do receive formal training in MC. It is unknown how medical students perceive MC, including its efficacy, appropriateness in medicine, its possible adverse effects, and its value for patients. This study investigated medical students’ perceived knowledge, beliefs, and attitudes toward MC to better understand their knowledge about and attitudes toward MC.

Method

Using a semi-structured interview guide, eight focus groups were conducted with 83 medical students via Zoom virtual meeting platform (Zoom Video Communications, Inc., San Jose, California, United States) in June 2022. The interviews were guided by the following content areas: (1) beliefs about cannabis' therapeutic utility, (2) perceived knowledge about MC, (3) the role of the physician regarding MC, (4) concern for cannabis’ adverse effects, and (5) MC education in the school curriculum. Data were analyzed using thematic analysis, an iterative, systematic process of coding patterns, and emerged themes in the interview data to explore medical students’ perceptions about MC. Themes were validated based on whether each theme captured distinct parts of the interview data and whether their content cohered meaningfully.

Results

Four themes emerged from the focus group interviews investigating medical students’ perceptions of MC: (1) erroneous beliefs about MC, (2) unreliable sources of information, (3) mixed attitudes toward legalization, and (4) desire for MC education while in medical school. Attitudes regarding MC in general, including legalization, varied by United States state of origin of the student and exposure to MC (e.g., use by family member).

Conclusion

MC seems to be a significant issue for medical trainees who might be required to recommend it to patients and manage coexisting therapies. Cultivating new knowledge about students’ perceptions and perceived knowledge about medicinal options and dosing of MC is critical for medical educators as they design undergraduate curricular initiatives for future physicians.

## Introduction

Recent attention has been given to the efficacy of medical cannabis (MC) for symptom management (e.g. as an anti-emetic and analgesic for pain) for post-traumatic stress disorder (PTSD), Crohn’s disease, ulcerative colitis, Alzheimer’s disease, multiple sclerosis, muscle spasms, and epilepsy among other medical conditions [1-15]. Well-controlled clinical trials indicate that MC can be an effective option (as a single or add-on treatment) in treating non-responding chronic pain in certain adults, but this type of evidence is lacking for other conditions [16-19]. 

Along with its potential therapeutic benefits, adverse effects from cannabis use have been documented, such as neurocognitive deterioration including memory impairment [15,16,20-25]. What is also likely is cannabis use in children and young adults carry a higher risk for long-term deficits [25]. In addition, smoking cannabis has been seen to produce carcinogens such as nitrosamines, polycyclic aromatic hydrocarbons, not unlike those carcinogens produced by smoking cigarettes [26]. Nonetheless, patients are using MC. By mid-2022, 36 states of the United States (US) had enacted effective MC laws, with an estimated 3.6-5 million state-legal MC users [27,28]. As the legalization of MC spreads across the US, more physicians - while not necessarily supporting cannabis use - are increasingly in support of effective regulation of it [27-29].

Most physicians know little about the therapeutic properties of MC (e.g., for which health conditions MC has been indicated effective) and less about recommending it (e.g., adverse effects, proper dosage) [29-31]. Many are also not equipped to answer questions from patients regarding its uses and possible adverse effects [32]. This may be due to the fact that physicians receive minimal or no education regarding MC during medical school [32,33] and that the US Drug Enforcement Administration (DEA) has classified cannabis as a Schedule 1 drug, which is the same legal, regulatory category as heroin (diacetylmorphine), LSD (lysergic acid diethylamide), mescaline (peyote), and MDMA (3,4-methylenedioxymethamphetamine or “ecstasy”) [34]. Schedule 1 drugs are those that are considered to have no substantiated medical value, a high potential for abuse, and the potential to create dependence [34]. 

There is a paucity of published research regarding preclinical and clinical training or practice experiences among physicians regarding whether to certify the patients for MC or not, dosing, and care management [35]. What we do know, however, is that physicians tend to rely on a combination of unreliable sources to obtain information, most of which are anecdotal reports [35,36]. 

Moreover, physicians perceive more research on MC is warranted, particularly for disease conditions such as cancer, chronic pain, and anxiety and for safety concerns such as the potential for addiction, dosing/product choice, effects of smoking/vaping, and medical contraindications such as drug interactions, and comorbidities [36]. Interestingly, physicians favor the legalization of MC more than medical students [37]. 

Due to the enduring stigma associated with MC, cannabis use is traditionally incorporated (if at all) in didactic lectures that discuss other Schedule 1 drugs and may only include its harmful effects as a “street drug.” Administrators may feel uncomfortable integrating the therapeutic uses of MC into their school’s curricula. By proxy, students could perceive cannabis in a negative light (i.e., as an illicit, harmful substance with no therapeutic value) and may judge future patients who find medical benefits from its use.

Medical students, as a rule, do not receive formal education related to recommending MC or managing its use [38]. In fact, one study of medical residents/fellows discovered that nearly 85% of medical schools or residency programs provided no MC education/training, and most reported they were ill prepared to answer questions from patients regarding MC [32]. In addition, communication skills regarding MC are not taught. These skills may be needed for an interchange between primary care/specialty physicians and those physicians who register their patients for MC use. Lack of communication between providers can expose patients to potential cannabis-drug interactions and other safety issues [32].

Some studies have investigated medical students’ attitudes and knowledge regarding MC and its use as a medical intervention [36,39-47]. Research from the US and other countries indicated that medical students possess a considerable desire for more formal education about MC, including learning about its potential for dependency/addiction [37,48]. Other published research indicates that medical students lack knowledge about MC and would not know how to counsel patients regarding its use [49,50].

Changes in the legalization of MC in the US are expected to go on and the debate over its effectiveness will continue. Integration of MC in the curricula may be warranted as students graduate and practice in states where MC is legal. It is still unknown how medical students in the US perceive MC, including its efficacy, appropriateness in medicine, its possible adverse effects, and its value for patients. The aim of this research was to assess medical trainees’ perceptions about MC to glean a better sense of (1) the gaps in their knowledge about MC, (2) any erroneous beliefs about the efficacy and/or harmful effects of MC, and (3) their desire for the integration of MC education in their school curriculum. This study is one of the first of its kind to use in-depth qualitative interviews with medical students to explore medical students’ perceptions toward MC for which little data are available. 

Research question

This qualitative study sought to answer the question: What do medical students know and think about MC? The specific aims of this study were to (1) conduct in-depth focus group discussions with osteopathic medical students to identify their attitudes, knowledge, and beliefs about MC and (2) analyze the data from the focus groups to identify emerged themes that may help guide new medical education curriculum strategies.

## Materials and methods

Overview

This qualitative study was designed to collect descriptive and conceptual findings through focus group interviews with medical students whose curriculum does not offer any MC training. Analyzing qualitative data allowed for the exploration of ideas and the collection of stories and experiences of participants to help further explain quantitative results from the published literature [51]. The goal of this study is thus to identify themes regarding MC that may be amenable to change through future curricular enhancements. This study was approved as a study with human subjects by the Nova Southeastern University Institutional Review Board (approval number 2022-155). The study was conducted at Nova Southeastern University, Fort Lauderdale, Florida, United States.

Procedures

Medical students at a school of osteopathic medicine of the University were recruited via an email listserv provided by the school’s office of student affairs. This included students who had just completed their first year (“rising” second-year students), just completed their second year (“rising” third-year students), and just completed their third year (“rising” fourth-year students), hereafter referred to as M2, M3, and M4 students. The email contained a cover letter with information about the study, that participation was strictly voluntary, and that participation (or non-participation) would have no bearing on their grades or standing in the medical school program. They were also informed that they would receive a $30 Amazon gift card (Amazon.com, Inc., Seattle, Washington, United States) in appreciation for their time. Those who expressed an interest in participating in the study were then contacted and scheduled to participate in one of the focus groups. All sessions were held in June 2022.

Using a semi-structured interview guide created by the researchers (Table 1), eight focus groups with a total of 83 medical students were conducted by the researcher via Zoom virtual meeting platform (Zoom Video Communications, Inc., San Jose, California, United States). The focus groups lasted between 60-75 minutes and were guided by the following content areas: (1) beliefs about cannabis' therapeutic utility, (2) perceived knowledge about MC, (3) the role of the physician regarding MC, (4) concern for cannabis’ adverse effects, and (5) desire for medical education regarding MC while in school. 

**Table 1:** Semi-structured Qualitative Interview Guide for Focus Groups

	Item
1.	Tell us what you know about the potential uses of medical cannabis.
2.	In what ways do you think medical cannabis has/does not have therapeutic value.
3.	Discuss how ready you feel you are to use cannabis in future practice.
4.	Discuss any concerns you have about the use of medical cannabis.
5.	What kind of education or curricular programming (if any) do you desire while in medical school? in residency?
6.	Discuss your beliefs if medical cannabis has the potential to be addictive or misused.
7.	Discuss your opinions about legalization of medical cannabis and recreational cannabis at the federal level.

Prior to starting the focus group, the nature of the study was discussed: its purpose, the voluntary nature of participation, confidentiality of the data collected from the interviews, possible risks/benefits, and plans for disseminating the results. Verbal consent was provided by all the participants in the focus groups before beginning the session. At the end of the session, each participant was emailed an Amazon gift card worth $30 for the one-time focus group interview in appreciation of their time.

Data analysis

Data saturation, the criterion for discontinuing data collection and/or analysis, was reached in the seventh focus group. However, to ensure the widest possible range of data on a category was obtained, an eighth group was added, yielding a final sample size of 83 participants [51,52].

Data were analyzed using thematic analysis, which utilizes systematic coding to detect patterns and themes to examine the perceptions of medical students regarding MC [53,54]. This process involves identifying segments of meaning contained in single words, phrases, or quotes in the text and cataloging these segments with codes that captured their meaning. Adhering to Braun and Clarke’s approach, the following data analysis stages were utilized: familiarization with data, generation of initial codes, search for themes among codes, review of themes, and defining and naming themes [52]. Two members of the research team read the interview transcripts and independently coded them. They then discussed the codes to confirm the accuracy and consistency of the coding of participants’ responses. Discrepancies were resolved through discussion between the researchers before identifying emergent themes.

Themes were then investigated by comparing across the data to consider different perspectives [52,53]. This thematic analysis was compared to the initial analysis and then developed into a final framework, focusing on perceptions of MC specific to medical students. This framework was discussed by all research team members (one of which was a medical student) from different disciplines with combined expertise in medical education, social work, public health, and behavioral health, each using their different perspectives to ensure the themes reflected the data. Data relating to experiences not associated with the topic of this study was not included in the final identified emerged themes.

Through this inductive approach, four themes emerged that captured an overall picture of the medical student experience without filtering them through a theory [52,53]. Themes were validated based on whether (1) each emerged theme captured distinct parts of all the interview data, (2) their content cohered meaningfully, and (3) each theme captured distinct parts of the overall data set [53].

## Results

The breakdown of the 83 participants according to the groups is as follows: M2 (n=43; 51.8%), M3 (n=18; 21.7%), and M4 (n=22; 26.5%). Due to the timing of the study (i.e., June 2022), incoming first-year students had not begun classes yet and therefore were not included.

Most of the participants held favorable views of reclassifying cannabis as a Schedule 1 drug to a lower classification for potential abuse and federal legalization of MC (albeit less favorable views for legalizing recreational cannabis). While some participants were able to recall medical conditions for which they thought MC could be therapeutic, few were able to report on health conditions for which MC would be contraindicated or have little or no benefit. They also had several concerns about the use of MC, including the potential for addiction, misuse (including giving or selling prescribed MC to others), and long-term adverse effects. Overall, participants reported that all the information they obtained about MC was received anecdotally (e.g., a family member used MC) or through social media (e.g., Twitter, Facebook, Instagram). The participants also reported that their medical school does not offer MC training or content in the course curricula (unless presented along with illicit, harmful drugs). Lastly, an overwhelming majority felt that MC education should be integrated into the medical school curriculum. 

The four themes that emerged from the focus group interview investigating medical students’ perceptions of MC are as follows: (1) erroneous beliefs about MC, (2) unreliable sources of information, (3) mixed attitudes toward legalization, and (4) desire for MC education while in medical school. Attitudes regarding MC, including legalization, its utility, and Schedule 1 status, varied by which state the student came from and if they had prior exposure to MC (e.g., family member used it). 

Erroneous beliefs about MC

Overall, participants reported what they thought were potential therapeutic uses of MC. The most common responses included cancer care, AIDS care, chronic disease management, pain management, digestive issues (e.g., irritable bowel syndrome), appetite stimulant, help with nausea, Parkinson’s disease, anxiety/depression, glaucoma, PTSD, sleep disorders, and multiple sclerosis. There was some consensus that MC may have a viable role in medicine, but there were many concerns regarding the potential harmful effects of MC. 

Student Concerns about MC Use

M2: Any concerns with lung issues, for example. Especially if it's consumed in the smoking form that would be something to think about, especially if the person has to keep taking it in order to ease their pain, there might be some long-term consequences to that.

M4: I read a paper several years ago on psychiatric conditions…regarding medical marijuana… they are more predisposed to actually developing full blown schizophrenia, and that would affect them negatively later on in life.

M4: Another concern would be people with anxiety…a lot of people recommend just chilling out with MC but it actually can evolve into a panic attack for certain people with anxiety so that'd be another concern.

M2: I am also concerned about using it for various mental illnesses like someone has something severe like bipolar disorder or schizophrenia…just not knowing how the chemicals are imbalanced and using marijuana, which we know has a very strong effect on how the brain works and brain chemistry.

M2: I think the biggest side effect I’ve heard about in our actual lectures to this point was cyclic vomiting syndrome…which I don't know very much about…but I have had some friends that really smoke a lot of pot, and they have gone to the hospital for like vomiting…just kind of uncontrollably.

Potential Addictive Quality of MC

Many participants believed that cannabis was a highly addictive substance, either physiologically or psychologically. They believed that addiction meant the “inability to function without the substance,” that one would “do whatever it takes to get that substance,” and that the person “cannot cope without that substance.” 

M4: It's like a gateway drug that you'll start using other substances when you start using marijuana or cannabis…In my own life, people that use cannabis are just like a drug addict.

M2: Medical marijuana or MC with other drugs is going to hurt the patient…and I still am concerned about addiction, and this will lead to other drug use. If you're getting it from a provider, maybe that lessens the risk, but I just know that it can be addictive…from what I've seen, I guess.

M2: I can definitely see this like a dependency or an addiction, where you reached out to cope with any form of anxiety or depression, even if it's used for milder mental health disorders, because there are definitely healthy ways to cope with that. We as medical students know what stress does to our brain and our body. I think if we're not careful about how we cope with that with physical exercise and social connection and other healthy ways to cope with stress, it can definitely lead to dependency because it's kind of a way to “put a band aid over it,” and then it happens again and you reach for it again, so that's a bit concerning as well.

Little Trust in Patients

Several participants commented that patients are not capable of managing their own MC use, such as not being in touch with their own bodies and/or illness. 

M2: It's (having a MC card) more patient driven so I feel like it could also have some type of negative effect on the patient as well if they don't know what they need for their condition or if they're not sure what they're feeling.

Some participants reported they thought there was potential for patients to misuse MC by giving it away or selling it to others for a profit. 

M2: It's obviously a concern with every medication amount…like the abuse of having that medical marijuana card. For purchasing for friends, they are creating a second tier of drug dealers…but it would just add to that level of drug trafficking in a way.

M2: I think it's the same as Adderall…it's kind of easy nowadays to get it prescribed even if you don't technically need it. I would only be concerned that people who don't necessarily need (MC) are getting it and maybe not even using it…trying to sell it…to other people who might not need it medically.

Participants were asked if they thought it was appropriate for physicians to recommend MC to patients via telehealth conferencing. While some participants were comfortable with it, others reported feeling that it should be done face to face.

M4: I feel like it opens up an opportunity for people to beat the system… I feel like that's like the big issue … like there's ways to get the medical card to receive the medical marijuana even if you may not actually have a chronic condition so there's just that opportunity for the system to be broken. Especially with telemedicine where they can have you see a doctor who doesn't really even know you, but you may have filled out an application. And you may not be actually telling the truth in the application, but I felt like there's just an opportunity for things to not be fully the truth.

M2: I would say it's (telehealth video conferencing) not appropriate if it's for psychiatric use…if you're using it for anxiety or depression or something like that, I think, in that sense, maybe an in-person evaluation would be better.

Confusion Between MC and Recreational Cannabis

Throughout the interview process it became apparent that when asked about MC, participants often spoke about recreational cannabis without distinguishing it from medically prescribed cannabis. In fact, the group facilitator had to remind participants in each of the eight focus groups at least twice to be mindful of this distinction, and to respond to the researcher’s questions with MC in mind and not recreational cannabis (unless specified). 

M2: I'm also pretty sure that when it comes to the prescription it's on a monthly basis, and then you can get like a certain amount, and I think it's a pretty large amount that you can get at once, depending on their prescription, but I know people have been able to get a very large amount at once, which I think could be a problem, because I know people that have used it recreationally and they say “I’m just like falling into like using it too much and it's kind of been counterproductive,” so I wouldn't want someone who has the prescription to fall into something like that.

Unreliable sources of information

When asked from where they got their information about MC, the participants reported anecdotally (e.g., family member, friend), social media platforms (e.g., Twitter, Instagram, YouTube), and news clips online. Only two of the 83 participants reported they searched the library databases or read scientific journal articles to obtain information about MC. In addition, the participants reported that their medical school does not provide any curricular-based education on the MC, but information is provided on the harmful effects of recreational cannabis (combined in a lecture with heroin, cocaine, and other illicit drugs). 

M4: I personally get the majority of my news (about marijuana) through Facebook. I follow various news companies and articles posted…that's how I get most of my news.

M4: This is the information that I’ve been hearing for at least six or seven years. While I was an undergrad, I would hear of case studies of this happening…I sometimes see videos, whether on YouTube or on other forms of social media.

M2: I think Twitter and Instagram probably are the two main ones where you would see a lot of this information from.

M2: Personally, I’ve never done any research on the topic, but I do know a lot of people who use MC, that's what I go off of to make my judgments, just like their experiences and how it's helped them either with their pain or with their nausea. So, it's mostly from observation for me.

Moreover, many participants commented that they believe there is little or no scientific research available on MC and that is why they rely on social media for information.

M2: I’ve always had the assumption that research is currently ongoing or that not a lot of data is out there currently.

M2: Sometimes I will see a study pop up on a social media platform and one of my first assumptions is that it probably doesn't have good controls, maybe there it's more of a survey style…they're asking people about how much they smoke and there probably are better studies, but I just haven't seen that…You know, medically controlled with just prescription marijuana.

M2: I'm under the impression that because it's still a Schedule 1 drug, federal funds cannot be used for research…so that's why there isn't a lot of research in the area, because only states can use their own research funds. That's why I’m under the impression there is not a lot of marijuana medical marijuana research.

Mixed attitudes toward legalization

The majority of participants agreed that MC should be federally legalized and also reclassified from being a Schedule 1 drug. Many were of the opinion that MC be rigorously controlled by physicians and no other health care provider (e.g., pharmacists, nurses) should be able to recommend/write orders for MC. Reasons cited for support for federal legalization include equal accessibility for all patients, proper screening process, the physician deciding what is best for the patient, standards will be in place, federal regulation will make the product safer, allowing for more research to be conducted, and benefits of MC outweigh its potential risks.

M2: I definitely think that it (MC) should be federal and equal across states. I have seen people that travel to Florida and move to Florida when they have chronic conditions where they find that marijuana is very therapeutic for them. They move to a state that has medical marijuana laws where it's approved versus living in a state that it's not…I think having equal accessibility throughout the entire country is really important.

M2: I believe that if there's a proper screening process then it (federal legalization) should be okay, because it would ultimately be in the physician’s best judgment to determine whether or not their particular patient would benefit from the medication and a dose frequency…as long as the physician is properly trained and there is a certification course that everyone has to take…it would give some kind of rule set for everyone to follow that'd be much easier to determine on an individual state by state basis or physician by physician basis, like a stricter rule book would be better, just because I do believe that marijuana could be could fall under the same kind of class of drugs that you would prescribe such as opioid so it's kind of like very addictive or very easily abuse medication, so I think having a better rule set for those kind of medications would be much better if it was nationwide.

M4: Cigarettes are legal and they're worse (than cannabis). We've seen that they (cigarettes) cause cancer and has addictive qualities and they’re legal, so I think legalization of marijuana should happen.

Many participants commented that cannabis should be reclassified from being a Schedule 1 drug. Some reasons for reclassification include low potential for addiction, medical benefits, and decreasing the stigma surrounding cannabis.

M2: I think that it (cannabis) should be removed as a schedule one drug…Needs to be revisited and perhaps remove it when revisiting what Schedule 1 actually means and what marijuana actually does.

M4: I think in terms of the scheduling, I don't really see a reason for it to be a Schedule 1 in the sense that it's in the same class as heroin and LSD.

M3: I don't think that it's necessarily a high potential for addiction….it's more like moderate and depends on certain factors of the person, and the reason for using it, so I wouldn't classify it as a Schedule 1 for the reason that it is proven to have been used in medicine and secondly that it's not necessarily a very high potential for addiction…it's lower than higher in my opinion.

M3: There has been promising research (in MC), but I think a huge drawback is that we can't move forward with it as much as we would like to because of that stigma.

Desire for MC education

As mentioned, no formal MC training exists in the participants’ medical school program. Cannabis is discussed in coursework only as it pertains to misuse, abuse, or risks associated with recreational use. Participants commented that their knowledge about MC was lacking and felt they would benefit from MC education while in school. 

M2: I just think the more knowledge, the better, and the more educated we are, the better we can help our patients, so even if we don't agree just having the knowledge about it to properly educate our patient, I think would be beneficial.

M2: I wonder if there's a benefit to having more formalized education…because sometimes when you just hear things from other people, you could just be hearing the wrong thing…Google searches that could be unreliable websites and controlling the information we're getting through school can make sure we're getting the facts…coming from current physicians who may be properly do the research first before telling us about it.

M2: I think, also having a background information in your didactic years and then going to rotations having some sort of information is going to help that rotation enhance your knowledge even more rather than going in completely blind. Even having a little bit of background information will always help.

The majority felt that MC education should be integrated throughout the four years of undergraduate training (i.e., didactic courses and clinical rotations). When asked about what they would like to see in an MC curriculum, participants reported that MC information (including therapeutic benefits, potential risks, and adverse effects, medical contraindications, legalities, and dosing recommendations) should be incorporated anywhere (and everywhere) in the curriculum but suggested it covered in pharmacology and/or systems courses.

M2: I think there's plenty of opportunities for us to be able to find ways to integrate MC in a number of lectures…professors can provide links to the citations they used…we're not being trained to become experts in MC, but we certainly should be familiar with what current literature is available. To be able to find a way to incorporate it into a GI lecture to incorporate segments of it and neurology into biochemistry…I think that's totally feasible in a pharmacology course.

M2: I think it should be just treated like any other drug…the treatment options….the adverse effects for those treatment options. This way, we are like lowering the stigma for it as well.

Some participants who had been on clinical rotations felt their preceptors were not amenable to teaching about MC. 

M4: My most recent preceptor said he was against medical marijuana. I asked him about it, and he said that there are so few applications for medical marijuana and legalizing medical marijuana would lead to other addictions, because it is a gateway drug, per his words, and I just kind of dropped it after.

M3: I've worked with one preceptor who was completely against medical marijuana because later after I spoke with him, he associated medical marijuana with recreational use. He put them on synonymous terms, so he was completely against it.

## Discussion

Four major themes emerged from interviews with 83 medical students during eight focus group sessions that included erroneous beliefs about MC, using unreliable sources to obtain information about MC, mixed attitudes about legalization and DEA reclassification of cannabis, and desire for MC education while in school. To our knowledge, this is the only published qualitative study conducted with medical students to investigate the beliefs and attitudes toward MC.

Erroneous beliefs and unreliable sources of information 

Almost all of the participants had heard somewhere of at least one thing MC might be used for (whether correct or incorrect); that is, they were able to mention one health condition associated with MC use. Their concern for MC’s potential for addiction took precedence over all other concerns (e.g., selling to others and the potential for long- and short-term adverse effects). However, most participants were not clear on the accurate definition of addiction or substance use disorder (SUD). Additionally, they often confused recreational cannabis use with physician-prescribed MC use in terms of addition and potential for adverse effects. There is limited if any qualitative exploratory published research about medical student MC knowledge and perceptions, making it difficult to ascertain if this confusion existed among medical students from other schools. It is difficult for quantitative surveys to depict such nuances. 

What was unsettling was the manner in which participants spoke about the dangers of MC use, that is, with unsubstantiated authority. Most believed MC was highly addictive and produces serious adverse effects (e.g., lung cancer, cyclic vomiting syndrome, schizophrenia, panic attacks, and cannabis-induced psychosis), but were unable to identify any research to confirm that or how they even came to know this "fact." Concerns about addiction to MC among medical students have been reported in previous studies [37]. The finding that students are uninformed about MC facts is supported by previous research with medical students [37,40,45-47]. They held different opinions about what the idea of addiction was but could not offer any medical or scientifically based definition or interpretation of what addiction was. Some called it a gateway drug to other drugs or felt that patients use cannabis to cope with stress instead of “exercising” and “making social connections.” 

Equally unsettling is the idea, according to participants, that patients are incapable of recognizing signs of their illness and knowing their own bodies, which could be dangerous to the patient as they “do not know what they need for their condition, or they do not know what they are feeling.” This belief can undermine the concept of patient activation, a measure of an individual's understanding, competence, and willingness to participate in care decisions and processes, which is a key component of treatment [55, 56]. Participants also felt that patients cannot be trusted, stating they will “falsify” applications for MC or purchase MC in bulk quantities to sell for profit equating it with “drug trafficking.” These erroneous beliefs are no less than disconcerting given (1) these students have had no formal training in MC, (2) are not likely to receive MC education while in their medical program, (3) rely primarily on social media for information about MC, (4) believe there is no or limited research on MC to guide treatment protocols, and (5) will soon be future physicians treating patients for whom MC may be a viable option for their healthcare needs. 

Legalization and cannabis classification 

Many students commented that cannabis should be reclassified from being a Schedule 1 drug. Some reasons for reclassification included low potential for addiction, medical benefits, and decreasing the stigma surrounding cannabis.

Paradoxically, while the majority of participants believed earlier in the discussion that MC was highly addictive with potentially dangerous adverse effects with little published research available on its therapeutic value, the majority also believed cannabis should be reclassified from being a Schedule 1 drug (i.e., substances with no current federally accepted medical use and a high potential for abuse) and that it should be federally legalized, which is supported by previous research conducted with physicians [27,28]. The idea that participants were in favor of legalizing MC aligns with their beliefs that (1) patients cannot be trusted, (2) MC should be in the hands of physicians (and only physicians), and (3) due to its “highly addictive” nature, MC should be prescribed and controlled in the same manner as opioids and other highly addictive medications. Some participants mentioned the idea that research regarding MC has been moving forward and may even be promising, but the stigma surrounding cannabis as a dangerous street drug inhibits this progression.

MC education

The medical school from which this sample was drawn, according to the participants, does not offer any curricular training in MC. Based on the results from the interviews, it was apparent that the participants have limited accurate information about MC and desire formal training while in medical school, a finding supported by previous research [37,45,46]. They recognized the benefit of having a formalized education regarding MC, and that Google searches and social media may be unreliable sources.

Of note is that participants were very interested in learning about MC’s therapeutic benefits, potential risks, and adverse effects, dosing recommendations, and the legal implications for physicians. They also recognized that they were not expected to be experts in MC but should be at minimum familiar with the current research and practice protocols. Participants suggested that MC education should occur during all four years of training. However, MC training during clinical rotations (years three and four) may be challenging as it would depend on the attitudes, comfort level, knowledge, and practice priorities of the preceptors in the field.

Another disconcerting finding of this study was that while participants, regardless of which year of the program they were in, (1) lacked accurate knowledge about MC and its potential adverse effects, (2) admitted to having no formal training nor education in MC, (3) relied heavily on anecdotal evidence and social media to obtain information about MC, and (4) felt confident that all their assumptions were correct and spoke with great conviction for their point of view. Medical educators may consider investing time in integrating MC content into their curricula to address these issues, as students will be entering graduate training positions in states where MC is legal.

Limitations of the study

Qualitative studies such as this cannot determine cause-and-effect relationships. The focus groups did not yield a representative sample, making it impossible to generalize findings to all medical students. Data for this study were collected from a sample of 83 osteopathic medical students from one institution in south Florida, a state in which MC is currently legal (although recreational cannabis has not been decriminalized); multi-site data collection from medical schools across the US may have yielded different results. Moreover, online internet focus groups may be different than in-person interviews with difficulties in determining which is an individual view and which is a “group” view [57]. There is the potential for researcher bias as well as participant bias as the medical students interviewed may have given a popular answer that their peers (or the group facilitator) seem to agree with rather than a true opinion, which can negatively influence the outcome of the study. Additionally, analysis and interpretation of the results are arduous for the researchers and serve as an additional limitation of focus group research [57]. Another limitation of qualitative methods is that patterns and trends are difficult to pinpoint. It is always advised to follow up with multi-site interviews and to perform a full-scope study using quantitative methods with large samples. 

## Conclusions

Overall, the medical students who participated in this qualitative study had little accurate knowledge about MC’s therapeutic uses and potential adverse effects, used unreliable sources for information about MC, but held favorable views of reclassifying cannabis from a Schedule 1 drug to a lower classification and felt it should be legalized on a federal level. Almost all participants felt that MC education should be integrated into the medical school curriculum. Laws governing MC use in the US will most likely change over time as MC’s popularity increases. Even in states where MC is illegal, the integration of MC content in medical school curricula may be prudent as graduate trainees may opt for residencies in states where MC is legal. Research studies continue to show the efficacy for medicinal use and proper dosing as well as the potential adverse effects of MC use, rendering accurate education important for medical programs to ensure MC readiness in future physicians.
